# The use of an interdental brush mitigates periodontal health inequalities: the Korean National Health and nutrition examination survey (KNHANES)

**DOI:** 10.1186/s12903-019-0858-6

**Published:** 2019-07-29

**Authors:** Jae-Young Lee, Hyun-Ju Park, Hyo-Jin Lee, Hyun-Jae Cho

**Affiliations:** 1 Department of Preventive & Social Dentistry, School of Dentistry, Seoul National University, 101 Daehakro, Jongno-gu, Seoul, 03080 South Korea; 2 Dental Research Institute, School of Dentistry, Seoul National University, Seoul, South Korea; 3 Department of Preventive and Social dentistry, Graduate School, Kyung Hee University, Seoul, South Korea; 4 Department of Dental Hygiene, College of Dentistry and Research Institute of Oral Science, Gangneung-Wonju National University, Gangeung, South Korea

**Keywords:** Interdental brush, Periodontal health inequality, KNHANES, Periodontitis

## Abstract

**Background:**

The purpose of this study was to evaluate the mitigating effect of the use of interdental brushes on periodontal health inequality.

**Methods:**

This study was based on the data acquired in the Sixth Korea National Health and Nutrition Examination Survey (KNHANES VI; 2013–2015). A total of 17,583 participants (7,633 males and 9,950 females)) aged 19 years or older completed the KNHANES VI between 2013 and 2015. Multivariable logistic regression analysis was performed using socioeconomic characteristics (sex, age, level of education, individual income), personal health practice (smoking, toothbrushing, dental flossing, interdental brushing, dental clinic visiting), systematic medical factors (diabetes mellitus, hypercholesterolemia, hypertension, obesity) and the community periodontal index. We confirmed differences in the prevalence of periodontal disease with the use of an interdental brushes stratified according to individual income.

**Results:**

Three logistic regression models adjusted for covariates hierarchically. In all models, individuals who used an interdental brush were not significantly different from individuals who did not use an interdental brush. The adjusted odds ratio (OR) for interdental brushing was 0.918 with a 95% confidence intervals (CIs) of 0.797–1.057. When periodontal disease was the outcome of the model, the lowest income group had 1.266 (95% CIs 1.066 to 1.502) times the odds of having periodontal disease than the highest income group. In interdental brush nonusers, the lowest income group had 1.276 (95% CI 1.061–1.533) times the odds of having periodontal diseases than the highest income group. However, in the interdental brush users, there were no significant differences in periodontal disease prevalence among income groups.

**Conclusions:**

The results suggest that the use of interdental brushes could alleviate periodontal health inequality.

## Introduction

Economic inequality can have a range of negative consequences for those people of low socioeconomic status (SES) [1]. Income and education level as indicators of SES are important factors that have profound impacts on oral health [2]. For example, several reports have shown that low SES groups exhibited worse oral health and a higher prevalence of oral diseases compared with high SES groups [3, 4]. Social gradients in oral health evidently exist at any time and place in the world: those with low SES exhibit a higher prevalence or greater odds of periodontitis than their counterparts with high SES [5].

The Global Burden of Disease Study, published by The Lancet, shows that severe periodontal disease is the sixth most common disease in the world [6]. Moreover, the prevalence of periodontal disease increased by 57.3% from 1990 to 2010, which makes it a global burden [7]. Periodontitis arises due to mature dental biofilm, which can easily remain in interproximal areas. As a recommendation for resolving this rapidly growing periodontal disease, the American Dental Association has emphasized self-care recommendations for interdental cleaning, which is probably the most universally applicable method [8]. According to a meta-review of interdental cleansing, the evidence suggests that interdental cleansing with interdental brushes is the most effective method for interdental plaque removal compared to dental floss, dental woodsticks, and oral irrigators [9]. The clinical efficacy of interdental brushes has been continuously studied, and the use of interdental brushes is recognized as a simple prevention method for periodontal disease [10–12].

The majority of studies on oral health inequalities were based on SES and environmental factors. A prior study revealed that socioeconomic status could be associated with a lower prevalence of dental caries and that water fluoridation reduced oral health inequalities [13]. Jung et al. [14] reported that because oral health habits developed during adolescence can persist throughout the course of a person’s life, intervention to address such inequalities in school environments are required.

As far as is known, however, there is no study about the relationship between the use of interdental brushes and periodontal health inequalities.

Therefore, the purpose of this study was to examine the prevalence of periodontitis between interdental brush users and nonusers. We hypothesized that interdental brush use would mitigate periodontal health inequalities in periodontal disease through comparison of prevalence rates of periodontal disease according to income difference between interdental brush users and non-users.

## Methods

This study used data acquired in the Sixth Korea National Health and Nutrition Examination Survey (KNHANES VI: 2013–2015). The KNHANES VI was a cross-sectional and nationally representative survey conducted by the Korea Centers for Disease Control and Prevention between 2013 and 2015. The target population of the survey was all non-institutionalized civilian Korean individuals 19 years of age or older. The survey employed stratified multistage probability sampling units based on the geographical area, gender and age, which were determined based on the household registries of the National Census Registry – the most recent 5 years national census in Korea. Using the census data, 200 primary sampling units (PSU) were selected annually across Korea [15]. Korea Centers for Diseases Control and Prevention (KCDC) Institutional review board approved this survey and written consent was obtained from all subjects without ethical approval because of the national survey design (IRB No. 2013-07CON-03-4C, 2013-12EXP-03-5C).

The minimum sample size to satisfy the study requirements was estimated at 1,953 adults. The assessment of association between periodontitis and socioeconomic covariates was estimated using the following parameters: 5% of standard error, 95% of power, 95% of confidence interval, odds ratio of at least 0.83 to be detected for logistic regression analysis [16]. The actual number of participants was larger than the minimum required by these parameters. Power curves were calculated using software (G*Power 3.1.3; Franz Paul, Universitat Kiel, Kiel, Germany) and indicate required minimum sample size needed at a range of power levels given logistic regression test [17].

The sampling protocol used was a complex, stratified, multistage probability cluster survey of a representative sample of the noninstitutionalized civilian population of Korea. A total of 17,583participants (7,633 males and 9,950 females), aged 19 years or older, completed the KNHANES VI in 2013 to 2015. From all the data collected by the KNHANES VI, we used the data on sociodemographic characteristics (age, sex, individual income, and educational level), personal health practice (smoking, toothbrushing, dental flossing, interdental brushing, and dental clinic visiting) and oral and general health status (community periodontal index [CPI], diabetes mellitus, hypercholesterolemia, hypertension, obesity) in the analysis. Use of interdental brush was used by main independent variable stratified by individual income for confirming differences in the prevalence of periodontal disease.

### Clinical examination

Clinical examinations were performed using mobile dental chairs and equipment. Oral examinations were conducted by trained dentists in compliance with the World Health Organization oral examination criteria. [18]. The mean interexaminer Kappa value was over 0.890 for tooth status and over 0.703 for periodontal status [19].

It was used the community periodontal index (CPI)_for diagnostic criteria of periodontitis. Periodontal conditions was examined out of six positions around the tooth (mesiobuccal, mesiolingual, distobuccal, distolingual, and mesial), except for the third molars. Participants who have CPI 3, 4 were considered having periodontal diseases.

### Interdental brushing using

The usage of an interdental brush was measured based on the validated Korean version of the oral health questionnaire. The main question is “Please select all products that apply to your oral health except toothpaste and toothbrush”. The health interview was conducted by trained health interviewers at a mobile examination center and at the homes of the study participants. Participants were asked about their use of an interdental brush, and categorized into two groups based on their interdental brush use: ‘nonuser’ and ‘user’.

### Covariates

The confounders of this study were the following major sociodemographic factors: sex, age, income, and education. The individual income was classified into four different groups: < 25% (the lowest quartile group), 25–49%, 50–74%, and 75–100% (the highest quartile group). Education level was also classified into four groups and was based on the Korean education system: below primary school, middle school, high school, and college or higher.

The personal health practices that were included in the analysis were: smoking status, daily toothbrushing status, the use of dental floss and whether the subjects visited a dental clinic within the last year. All of these indicators are nominal categorical variables.

The systematic medical factors included in the analysis were diabetes mellitus, hypercholesterolemia, hypertension, and obesity. With respect to diabetes mellitus, participants were classified into three groups defined as: a fasting plasma glucose level ≥ 126 mg/dL, a previous diagnosis of diabetes by physician, or current use of anti-diabetic agents or insulin. Hypercholesterolemia was defined as a total plasma cholesterol level of ≥240 mg/dL or current use of cholesterol-lowering agents. Hypertension was defined as an average SBP/DBP ≥ 140/90 mmHg or the use of anti-hypertensive agents. Based on the World Health Organization redefined criteria for obesity in the Asia–Pacific region, obesity was defined as a BMI of ≥25 kg/m^2^.

### Statistical analysis

All statistical analyses were conducted using SPSS version 23.0 (SPSS, IBM, NY, USA). All data were weighted in the statistical analyses to account for the complex multistage, stratified, and unequal selection probabilities or clustered sampling design associated with the KNHANES VI. Appropriate sample weights were selected as specified from each national data set.

Generalized linear models and chi-square tests were used to compare the complex sample survey data characteristics of subjects in the interdental brush user and nonuser groups. Multivariable logistic regression models were used to identify associations between using an interdental brush and periodontitis after adjusting for potential confounders. Logistic regression was used to calculate the odds ratios (ORs) with 95% confidence intervals (CIs) for periodontal diseases. All models were adjusted for sex, age, level of education, individual income, smoking, toothbrushing, dental flossing, dental clinic visiting, diabetes mellitus, hypercholesterolemia, hypertension, and obesity. Regression model 1 adjusted for age, sex, individual income and level of education. Personal health practice (smoking, toothbrushing, dental flossing, dental clinic visiting) was added to regression model 2. Systematic medical factors (diabetes mellitus, hypercholesterolemia, hypertension, obesity) were added to regression model 3. Other multivariable logistic regression analyses were performed to identify the association between periodontitis and socioeconomic status after adjusting for potential confounders in the whole group, i.e., the interdental brush user and nonuser groups. A *P* < 0.05 was considered to be statistically significant.

## Results

### Demographics and clinical characterization

Table 1 shows the demographics and clinical conditions of the individuals included in this study, stratified by interdental cleaning users and nonusers. A total of 17,583 subjects were included in this study; 9,950 subjects were female (56.6%), and 7,633 subjects were male (43.4%). In Korea, 20.4% of individuals use interdental brushes.

**Table 1:** Characteristics of the study population stratified by interdental brush use

Variables	Interdental brush use				*P-*value
	Nonusers		Users		
	Unweighted N	Weighted % (95% CIs)	Unweighted N	Weighted % (95% CIs)	
Age (Mean ± SD)	51.72 ± 16.99		46.67 ± 14.84		< 0.001
Sex					< 0.001
Male	6,399	50.5 (49.6–51.3)	1,234	45.0 (43.1–47.0)	
Female	7,892	49.5 (48.7–50.4)	2,058	55.0 (53.0–56.9)	
Education					
≤ Elemental school	4,002	21.8 (20.8–22.8)	477	11.3 (10.2–12.6)	< 0.001
Middle school	1,970	13.6 (12.9–14.4)	311	8.3 (7.3–9.3)	
High school	4,151	34.6 (33.4–35.7)	1,138	37.7 (35.6–39.8)	
≥ University or college	3,621		1,245	37.7 (35.6–39.8)	
Income					
Low	3,566	25.7 (24.3–27.1)	687	21.4 (19.6–23.4)	< 0.001
Middle low	3,619	25.5 (24.3–26.8)	788	24.8 (22.8–26.8)	
Middle high	3,576	24.5 (23.3–25.8)	850	25.8 (23.9–27.9)	
High	3,455	24.3 (22.7–25.9)	952	28.0 (25.7–30.5)	
Smoking					0.883
Everyday	2,481	21 (20.1–21.9)	544	20.8 (19–22.6)	
Occasionally	2,637	18.1 (17.4–18.9)	563	17.8 (16.3–19.4)	
Never	9,170	60.9 (60–61.8)	2,184	61.4 (59.4–63.4)	
Flossing^a^					< 0.001
No	11,753	81.2 (80.3–82.1)	2,389	71.6 (69.6–73.5)	
Yes	2,538	18.8 (17.9–19.7)	903	28.4 (26.5–30.4)	
Daily Toothbrushing					0.013
No	289	1.7 (1.5–1.9)	40	1.0 (0.7–1.5)	
Yes	13,998	98.3 (98.1–98.5)	3,252	99.0 (98.5–99.3)	
Dental clinic visiting^b^					< 0.001
No	7,431	52.9 (51.8–54.1)	1,369	43.4 (41.3–45.4)	
Yes	6,850	47 (45.9–48.2)	1,923	56.6 (54.6–58.7)	
Diabetes^c^					< 0.001
Normal	7,244	68.4 (67.3–69.6)	1,950	72.3 (70.3–74.2)	
Impaired fasting glucose	2,563	22.0 (21.0–23.0)	591	20.8 (19.1–22.6)	
Diabetes	1,320	9.6 (8.9–10.3)	246	6.9 (6–8)	
Hypercholesterolemia^d^					0.840
Normal	9,238	85.7 (84.9–86.4)	2,320	85.8 (84.3–87.2)	
Abnormal	1,832	14.3 (13.6–15.1)	468	14.2 (12.8–15.7)	
Hypertension^e^					< 0.001
Normal	5,530	50.3 (49–51.6)	1,589	56.3 (54.1–58.5)	
Prehypertension	2,856	23.5 (22.5–24.5)	682	23.9 (22.1–25.8)	
Hypertension	3,967	26.2 (25.2–27.3)	718	19.8 (18.2–21.5)	
BMI related^f^					0.695
Underweight	548	4.8 (4.3–5.2)	135	4.8 (4.0–5.8)	
Normal	8,026	63.0 (62.0–64.0)	1,940	62.0 (60.0–64.0)	
Obese	4,166	32.2 (31.3–33.2)	1,012	33.1 (31.2–35.2)	
Periodontitis^g^					0.001
Normal	8,967	73.6 (72.2–74.9)	2,231	76.1 (73.7–78.3)	
Periodontitis	3,714	26.4 (25.1–27.8)	772	23.9 (21.7–26.3)	

^a^Flossing was used usually

^b^Dental clinic visit within a year

^c^Impaired fasting glucose was defined by 100 mg/dL ≤ Fasting blood glucose<126 mg/dL and diabetes was defined by fasting blood glucose≥126 mg/dL or drug or insulin

^d^Hypercholesterolemia was defined by total cholesterol≥240 mg/dL or drug

^e^Prehypertension was defined by 140 mmHg>Systolic blood pressure ≥ 130 mmHg or 90 mmHg>diastolic blood pressure ≥ 85 mmHg and hypertension was defined systolic blood pressure ≥ 140 mmHg or diastolic blood pressure ≥ 90 mmHg or drug

^f^Underweighted was defined by BMI<18.5 and obese was defined by BMI ≥ 25

^g^Periodontitis was defined community periodontal index codes 3, 4

There are many statistically significant differences between the interdental brush users and nonusers with regard to socioeconomic status, personal health practice, and systematic medical factors (Table 1). The main differences between the groups were that interdental brush users compared to nonusers were more likely to be female, have higher levels of education and income, were less likely to be smokers, and had more frequent dental visits (Table 1).

### Interdental brush use and periodontal diseases

Table 2 shows the results of the hierarchical regression analyses that determine whether there is a multivariable association between the use of interdental brushes on periodontal disease prevalence. The three logistic regression models were designed to hierarchically adjust for covariates. In all the models, individuals who used an interdental brush were not significantly different from the individuals who did not. In model 3, The adjusted OR for using an interdental brush was 0.918 with a 95% CIs of 0.797–1.057.

**Table 2:** Multivariable association between interdental brush use and periodontitis

	Odds ratio (95% confidence interval)		
	Model 1	Model 2	Model 3
Use of an interdental brush	*N* = 13,625	N = 13,621	*N* = 12,427
Nonusers	0.928 (0.814–1.058)	0.911 (0.797–1.042)	0.918 (0.797–1.057)
Users	Reference	Reference	Reference

Response variable: Periodontitis

Explanatory variable: Use of an interdental brush

Model 1 was adjusted to socioeconomic status variables (sex, age, level of education, individual income)

Model 2 was additionally adjusted to personal health practice variables (smoking, toothbrushing, dental flossing, dental clinic visiting)

Model 3 was additionally adjusted to systematic medical factor variables (diabetes mellitus, hypercholesterolemia, hypertension, obesity)

Table 3 shows the results of the logistic regression analyses for multivariable associations between periodontitis and socioeconomic status in the interdental brush user and nonuser groups after further adjusting for personal health practice and systematic medical factors. When periodontal disease was the outcome of the model, the lowest income group had 1.266 (95% CIs 1.066 to 1.502) times the odds of having periodontal disease than the highest income group. In interdental brush nonusers, the lowest income group had 1.276 (95% CIs 1.061–1.533) times the odds of having periodontal disease than the highest income group. However, in the interdental brush users group, there were no significant differences in periodontal disease prevalence among income groups. In group of 19 to 64 years old, there is also difference in the prevalence of periodontitis stratified individual income between interdental brush user and non-users. However, in group over 65 years old, it is not confirmed the difference in the prevalence of periodontitis stratified individual income between interdental brush user and non-users.

**Table 3:** Multivariable association between individual income and periodontitis in the entire sample, stratified by interdental brushing behavior and age

	Odds ratio (95% confidence interval)		
	Total	Interdental brush use	
		Nonusers	Users
Model 1	N = 13,625	*N* = 10,904	*N* = 2,721
Low	**1.347 (1.148–1.581)**	**1.357 (1.145–1.608)**	1.318 (0.945–1.838)
Middle low	**1.244 (1.076–1.438)**	**1.196 (1.022–1.399)**	**1.391 (1.018–1.901)**
Middle high	1.059 (0.918–1.222)	1.054 (0.898–1.236)	1.069 (0.781–1.464)
High	Reference	Reference	Reference
Model 2	N = 13,621	N = 10,901	N = 2,720
Low	**1.258 (1.070–1.478)**	**1.255 (1.056–1.492)**	1.240 (0.890–1.727)
Middle low	**1.175 (1.015–1.361)**	1.139 (0.971–1.337)	1.345 (0.980–1.844)
Middle high	1.027 (0.888–1.187)	1.031 (0.878–1.212)	1.010 (0.739–1.383)
High	Reference	Reference	Reference
Model 3	N = 12,427	*N* = 9,883	N = 2,544
Low	**1.266 (1.066–1.502)**	**1.276 (1.061–1.533)**	1.193 (0.839–1.696)
Middle low	1.154 (0.986–1.352)	1.113 (0.934–1.326)	1.351 (0.976–1.868)
Middle high	1.057 (0.907–1.232)	1.074 (0.906–1.274)	1.011 (0.736–1.390)
High	Reference	Reference	Reference
Model 3 (19–64 yrs. group)	N = 9,843	*N* = 7,609	N = 2,234
Low	**1.276 (1.050–1.549)**	**1.301 (1.052–1.608)**	1.154 (0.793–1.681)
Middle low	1.160 (0.965–1.394)	1.120 (0.914–1.372)	1.324 (0.931–1.883)
Middle high	1.055 (0.881–1.263)	1.076 (0.879–1.316)	1.001 (0.704–1.422)
High	Reference	Reference	Reference
Model 3 (over 65 yrs)	N = 2,584	N = 2,274	*N* = 310
Low	1.109 (0.830–1.483)	0.988 (0.967–1.011)	1.392 (0.566–3.425)
Middle low	1.064 (0.808–1.402)	1.062 (0.781–1.444)	1.688 (0.806–3.532)
Middle high	1.058 (0.821–1.363)	1.058 (0.805–1.391)	1.009 (0.530–1.921)
High	Reference	Reference	Reference

Response variable: Periodontitis

Explanatory variable: Individual income

Model 1 was adjusted to socioeconomic status variables (sex, age, level of education)

Model 2 was additionally adjusted to personal health practice variables (smoking, toothbrushing, dental flossing, dental clinic visiting)

Model 3 was additionally adjusted to systematic medical factor variables (diabetes mellitus, hypercholesterolemia, hypertension, obesity)

*P*-values highlighted in bold are statistically significant (*p* = 0.05)

## Discussions

Previously, there have been many studies on health inequalities due to socioeconomic factors and inequality in oral health. Celeste et al. [20] reported that greater municipal income inequality was associated with worse oral health even after controlling for individual-level variables. Additionally, Hobdel et al. [21] reported on the striking degree to which SES variables individually account for differences in three oral diseases in different countries, and chronic destructive periodontitis has a strong discernable association with SES variables. Mejia et al. [22] reported that individuals from lower income and education groups consistently experienced higher burdens of untreated dental decay and poorer self-rated oral health. However, papers, such as this one, that analyze the mitigation effect of oral health inequalities are very rare.

In this representative cross-sectional sample of Korean adults aged ≥19 years, the use of an interdental brush was associated with the mitigation of periodontal disease outbreaks. In our study, the use of an interdental brush showed an alleviating effect on periodontal health inequality. Multivariable analysis by hierarchical regression model demonstrated that using interdental brush alleviates periodontal health inequality, because interdental brush user showed no significant difference of periodontitis prevalence between socioeconomic status. Especially, non-interdental brush user who was lowest SES in 19–64 year age group, showed higher risk of 30.1 percentage comparing to highest SES group. Periodontal disease is the sixth most common disease in the world [23]. Globally, gingival bleeding is the most prevalent sign of disease, whereas the presence of deep periodontal pockets (≥6 mm) varies from 10 to 15% in adult populations [24]. In Korea, the number of periodontal disease patients increased from 7 million people to 11 million people in 2016, a 56.6% increase over the previous 4 years [25]. Additionally, according to frequent outpatient disease reporting in Korea, gingivitis and periodontal disease are the second most common diseases overall, and the majority of people are burdened with periodontal disease [26]. The burden of these diseases causes a loss of social resources, and even though there is variation in each country, treating oral diseases requires a tremendous amount of financial resources, and oral health inequalities in the treatment of oral diseases due to income may inevitably occur [27]. Not only is periodontal disease related to systemic diseases, but the transition to systemic diseases adds to the burden of other diseases [28–30]. In the FDI Tokyo Declaration of 2014, there was announcement for an action plan for future dental institutions and dental personnel through the World Periodontal Disease Initiative [31]. Thus, periodontal disease is recognized as a serious disease like other systemic diseases, and the need for global management and treatment is emphasized.

There are many methods to remove dental plaque and prevent periodontitis, including proper toothbrushing and dental flossing, brushing with dental woodsticks or interdental brushes, and using rubber-tip stimulators, irrigating devices and antimicrobial agents [32]. Previous studies have reported that interdental toothbrushes among these methods reduce periodontal disease and reduce interproximal tooth caries and missing teeth. [33].It is important to consider other factors as most interdental clean-up methods have different effects depending on the patient’s ability and willingness. [34]. Dental floss is the only tool that can reach into the interdental papillae. Hence, an interdental brush should fit in the interdental spaces if interdental papillae recede. Slot et al. [35] reported a meta-review that showed that, compared with floss, the majority of the studies presented a positive significant difference in the plaque index when using an IDB. In our research, there were no significant differences in periodontitis prevalence between interdental brush users and nonusers (Table 2). However, another study showed a positive effect of using an interdental brush with respect to plaque scores, bleeding scores and probing pocket depths [36–39]. The reasons behind the positive effect are the usage rate and the quality. In Korea, the interdental brush usage rate is only 20.4%, while in the US, it is approximately 68.9%. Additionally, the Korean questionnaire did not include detailed items related to frequency, period, or time. However, when stratified by individual income quartile, there were differences in the prevalence of periodontitis between the lowest income group and the highest group (Table 3). On the other hand, we were able to observe that there were no differences in periodontitis prevalence due to income in the group who uses interdental brushes. In addition, we were able to observe an increase in the OR accompanying the addition of covariate factors (Table 3).

In light of past studies that demonstrated the impact of income on health inequalities [40, 41], it was possible to reduce the disparity among groups having different socioeconomic status in prevalence rates of illness. The use of an interdental brush could alleviate periodontal health inequality.

The limitation of this study is that there are no specific questions regarding the use of interdental brushes like frequency and duration of use. Also, in this study, as a cross-sectional study, using interdental brushes did not allow analysis of direct effects to periodontal diseases. And it may also lead to information bias in response to self-reported questions.

However, in this study, a large sample analysis was conducted to represent the entire Korean population. In addition, many randomized controlled trial researches reinforced to relation between interdental brushes use and periodontal diseases on the individual level. Therefore, prospective follow-up studies may provide scientific evidence to support the beneficial effects of using interdental brushes. Another apparent limitation of this study is the use of CPI code to define periodontitis. We defined periodontitis as CPI scores of 3 or 4, classifying participants into two groups of non-periodontitis and periodontitis. We did not classify the subjects according to the severity of periodontitis and included both the shallow and deep periodontal pocket into a periodontitis group. Because national surveys in Korea use the CPI index that WHO uses for cross-country comparisons, and define periodontal diseases based on these criteria to classify periodontal conditions.

## Conclusions

Interdental cleaning is very important to prevent periodontitis. Interdental brushes are known to be the most effective among many periodontitis management methods. Our study supported the hypothesis that interdental brush use reduces the periodontal health inequalities by socioeconomic status.. Additionally, we found that the use of an interdental brush could alleviate periodontal health inequality.

## Data Availability

Sixth KNHANES data can be accessed and downloaded from the KNHANES homepage (URL: https://knhanes.cdc.go.kr/knhanes/eng/index.do).

## Abbreviations

BMI: Body mass index
CIs: 95% confidence intervals
CPI: Community periodontal index
DBP: Diastolic blood pressure
KNHANES VI: Sixth Korea National Health and Nutrition Examination Survey (2013–2015)
OR: Odds ratio
SBP: Systolic blood pressure
SES: Socioeconomic status
WHO: World Health Organization
